# Supplementary material for: Cloning and Characterization of Fructose-1,6-Bisphosphate Aldolase from Euphausia superba
Source: Int J Mol Sci. 2022 Sep 9;23(18):10478. doi: 10.3390/ijms231810478 (PMC9499490; doi:10.3390/ijms231810478)
Supplement: Supplementary file 1 [file ijms-23-10478-s001.zip › Supplementary material.pdf]

Table S1. Primer sequences

**Primer**	**Sequence(5'-3')**
3C-EsFBA-F	atggatgaactgtataaattggaggttttgttccagggtc
EsFBA-R	gtggtggtggtgctcgagtgcgtagtctggcacgtcgt
sfGFP-F	aaggagatataccatggtgagcaagggcgagga
SfGFP-3C-R	ctggaacaaaacctccaatttatacagttcatccatgccca

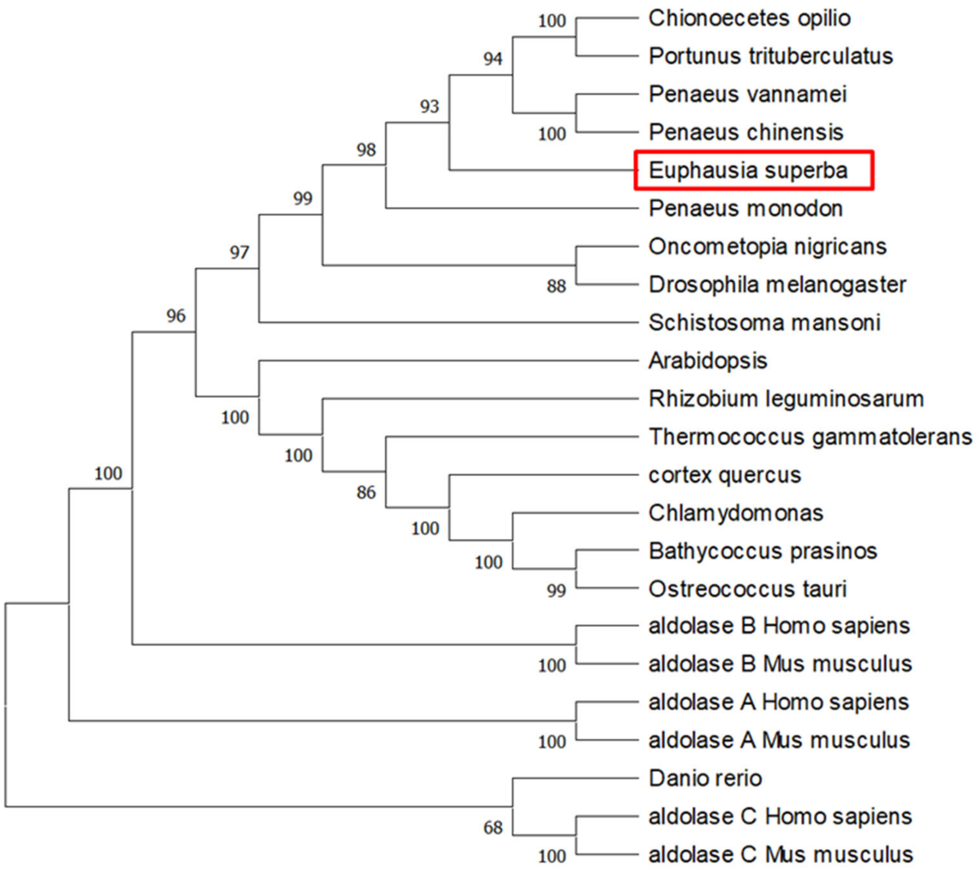

Figure S1. The phylogenetic tree constructed according to the sequences of EsFBA and other FBA.

EsFBA researched in this study is in red box.

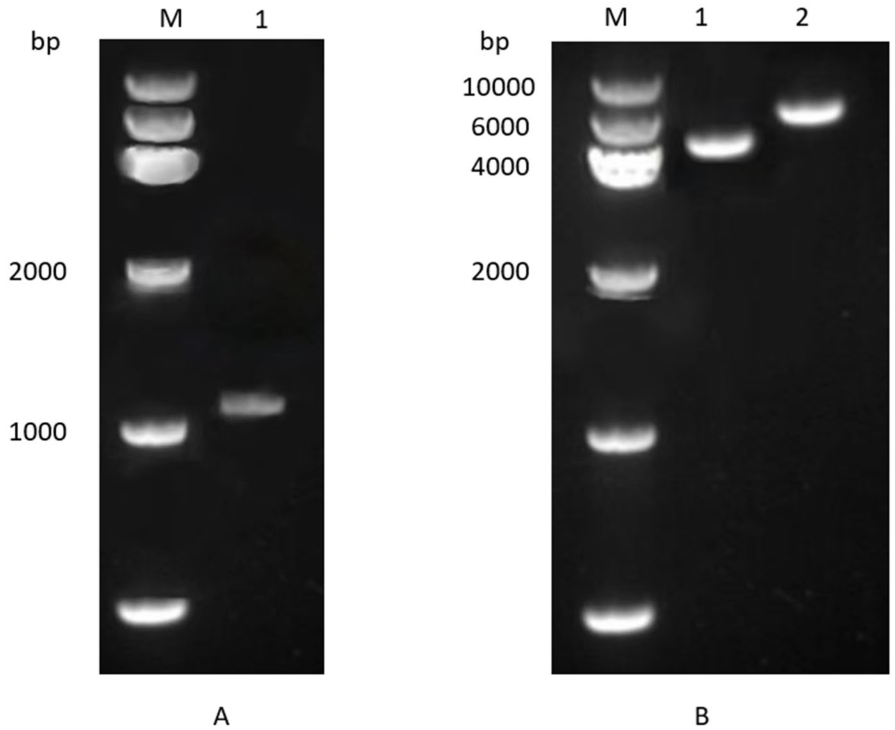

Figure S2. Agarose gels for testing recombinant expression vector constructs

A: M: marker; 1: PCR amplification product

B: M: marker; 1: Plasmid pET-28a; 2: Positive clone of pET-28a and target fragment.

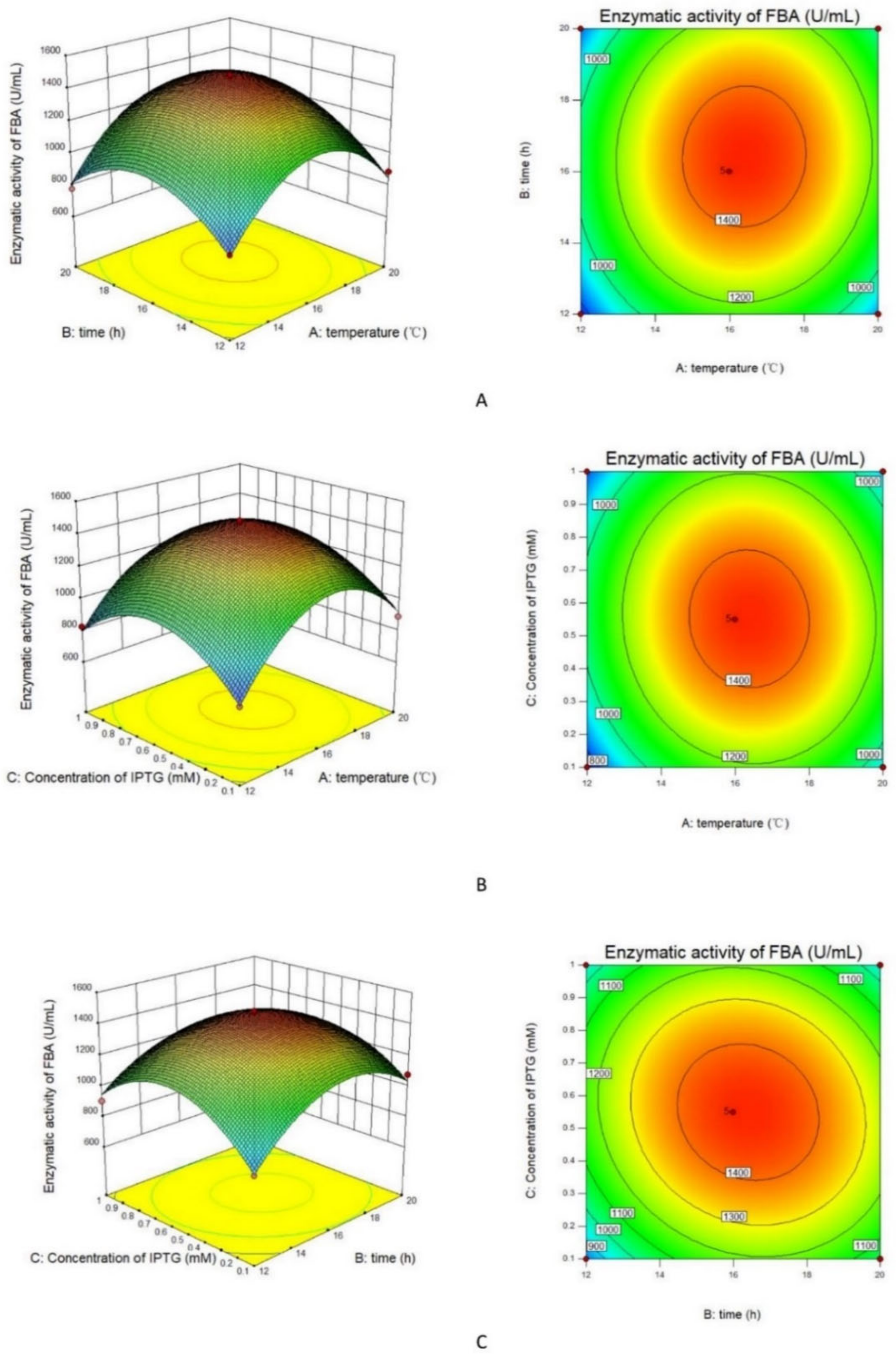

Figure S3. Response surface diagram and contour surface diagram of the interaction between factors on EsFBA enzyme activity.

A: Response surface relationship between temperature and time; B: Response surface relationship between temperature and ITPG concentration; C: Response surface relationship between time and ITPG concentration.
